# Topical Delivery of Atraric Acid Derived from *Stereocaulon japonicum* with Enhanced Skin Permeation and Hair Regrowth Activity for Androgenic Alopecia

**DOI:** 10.3390/pharmaceutics15020340

**Published:** 2023-01-19

**Authors:** Sultan Pulat, Laxman Subedi, Prashant Pandey, Suresh R. Bhosle, Jae-Seoun Hur, Jung-Hyun Shim, Seung-Sik Cho, Ki-Taek Kim, Hyung-Ho Ha, Hangun Kim, Jin Woo Park

**Affiliations:** 1 College of Pharmacy, Sunchon National University, Sunchon-si 57922, Jeonnam, Republic of Korea; 2 Department of Biomedicine, Health & Life Convergence Sciences, BK21 Four, Biomedical and Healthcare Research Institute, Mokpo National University, Muan-gun 58554, Jeonnam, Republic of Korea; 3 Korean Lichen Research Institute, Sunchon National University, Sunchon-si 57922, Jeonnam, Republic of Korea; 4 College of Pharmacy and Natural Medicine Research Institute, Mokpo National University, Muan-gun 58554, Jeonnam, Republic of Korea

**Keywords:** atraric acid, topical delivery, skin penetration, dermal papilla cell proliferation, androgenic alopecia, hair regrowth

## Abstract

Atraric acid (AA) is a phenolic compound isolated from *Stereocaulon japonicum* that has demonstrated anti-androgen properties and was used to design an alternative formulation for the treatment of alopecia. This new topical formulation was designed using a solvent mixture system composed of ethanol as a volatile vehicle, oleic acid as a permeation enhancer, and water for skin hydration. The ideal topical AA formulation (AA–TF#15) exhibited an 8.77-fold higher human skin flux and a 570% increase in dermal drug deposition, compared to 1% (*w*/*w*) AA in ethanol. In addition, compared to other formulations, AA–TF#15 (1% [*w*/*w*] AA) activated keratinocytes and human dermal papilla cell proliferation at a concentration of 50 µM AA, which is equivalent to 50 µM minoxidil. Moreover, AA–TF#15 treatment produced a significant increase in hair regrowth by 58.0% and 41.9% compared to the 1% (*w*/*w*) minoxidil and oral finasteride (1 mg/kg)-treated mice. In addition, AA–TF#15 showed a higher expression level of aldehyde dehydrogenase 1, β-catenin, cyclin D1, and pyruvate kinase M2 proteins in the skin of AA–TF#15-treated mice compared to that of those treated with minoxidil and oral finasteride. These findings suggest AA–TF#15 is an effective formulation for the treatment of scalp androgenic alopecia.

## 1. Introduction

Androgenic alopecia is characterized by the abnormal loss of hair, primarily on the scalp, of men at various ages [1]. A variety of factors associated with hair loss are due to changes in the hair cycle, such as hormonal imbalances; aging; nutritional supplement deficiencies; medications; genetics; and dermal complications, including fungal infections, which can cause alopecia [2,3,4]. Hair loss due to hormonal changes associated with puberty or loss observed in young subjects has been a recent concern worldwide [5]. An overabundance of circulating androgens, such as testosterone or 5α-dihydrotesterone (DHT; a converted metabolite of testosterone synthesized by 5α-reductase and primarily found as Type I or II isomers), leads to binding at the androgenic nuclear hormone receptors present in hair follicles, which activates the receptors [6,7]. As a result, the dermal papilla of hair follicles secretes numerous cytokines, such as transforming growth factor-β1, interleukin-1α, and tumor necrosis factor-α, which can shorten the duration of the anagen phase, enhance apoptosis of hair follicles, miniaturize hair follicles, and lead to hair loss [8,9,10,11]. After hair loss, the kenogen phase of the hair cycle was elongated for cases of androgenic alopecia, which may cause permanent hair loss [11]. Although alopecia is not a life-threatening disease, it can affect physical appearance, self-esteem, and confidence, which directly affects psychological health and social wellbeing [12]. Thus, the treatment of alopecia is important for maintaining and establishing quality of life.

While androgenic alopecia is a very common problem that affects quality of life, treatments are limited to drugs approved by the US Food and Drug Administrative (FDA) and include topical application of minoxidil and oral administration of finasteride. Other therapies, such as hair transplantation, injection of growth factors, and treatment with platelet-rich plasma have limited success in treating androgenic alopecia due to poor adherence and severe associated complications [13,14]. Topical application of minoxidil prolonged the anagen phase in dermal papilla by inducing vascular endothelial growth factor (VEGF) and the β-catenin pathway, and also stimulated follicular proliferation and differentiation [15]. However, minoxidil can only accelerate the growth of hair follicles, but not prevent hair loss. It is also reported to feel greasy and itchy due to the excipients in minoxidil solutions, such as propylene glycol. Dermatitis and headaches were common side effects of the application of marketed 5% minoxidil, which led to poor compliance [16,17,18].

The oral administration of dutasteride and finasteride are 4-aza steroids that act as competitive inhibitors of 5α-reductase enzymes to prevent hair loss. These two drugs can inhibit potent androgen-binding steroids (DHT) from being converted to testosterone, which enables the control of hair loss. However, testosterone still has some affinity toward androgenic binding receptors, which requires long-term treatment with dutasteride and finasteride at a high dose [19]. DHT is an important physiological component in the body that allows for nominal functioning and the development of organs, such as the reproductive system, testes, muscles, liver, skin, nervous system, and immune system [20]. Therefore, the use of dutasteride and finasteride may not be effective in cases of alopecia occurring in early adulthood, when the body is still developing. The long-term use of these drugs affects the normal physiology of the body and may elicit various side effects, such as reduced sex drive, ejaculation disorder, or impotence [21,22]. Thus, there is increasing demand for drugs with alternative mechanisms for the treatment of hair loss that can promote hair regrowth with high efficacy and with few side effects.

Atraric acid (AA; methyl 2,4-dihydroxy-3,6-dimethylbenzoate) is a naturally occurring phenolic compound that acts as a selective antagonist of the androgen receptor. AA competes with androgens, such as testosterone and DHT, for binding to androgen receptors and specifically inhibits androgenic receptor-mediated transactivation by reducing androgenic receptor translocation to the nucleus [7]. Therefore, the mechanism of AA may treat hair loss without affecting the balance of related steroid hormones, such as DHT, which are required for the development and normal functioning of the body. Besides the hormonal factors, AA can control several androgen-inducible negative mediators, including transforming growth factor-β, dickkopf-1, and interleukin-6.

The unique mechanism of AA was assumed to allow for normal biological activities related to hair follicles in androgenic alopecia [23,24]. The phytochemical constituents of *Allium ascalonicum* exert anti-inflammatory and anti-androgenic responses similar to those observed during hair regrowth by AA, including upregulation of genes associated with β-catenin and angiogenesis (VEGF) pathways [23,25,26]. *Serenoa repens* extracts also have anti-androgenic properties and prevent hair loss by blocking androgenic receptors, and promote hair regeneration by activating transforming growth factor-beta and mitochondrial signaling pathways [27]. These findings support the idea that AA, which was reported to have anti-androgen and anti-inflammatory properties, has the ability to be an effective treatment against hair loss in androgenic alopecia while also promoting hair regrowth. However, the effectiveness of any drug depends upon its availability at the desired location in the body. Therefore, the topical application of AA with a vehicle system that can enhance the permeability of the drug at the targeted site may be the best approach.

The widespread use of topical systems may limit the effective delivery of drugs due to the low water solubility of drugs, as well as the impermeable nature of the outermost layer of the skin [28,29]. These limitations may be addressed by using micro-structured systems, such as microemulsions, pastes, creams, ointments, gels, and nanoparticles, which have been investigated for their ability to improve drug solubility and delivery efficacy [30,31,32,33]. However, the remaining residual components in ointments, pastes, and creams can limit the continuous permeability of a drug and also reduce patient compliance [34,35]. Thus, a simple topical solution containing volatile and non-volatile solvents that effectively solubilizes drugs and maintains supersaturation of the drug at the applied area over time would be the best approach. This type of treatment would significantly increase the thermodynamic driving force, to promote permeability across the human scalp [36,37].

An effective permeation enhancer can facilitate lipid extraction from the lipid bilayer matrix, fluidization of the lipid bilayer, alteration of stratum corneum (SC) protein conformation, co-permeation of drug with alcohol or volatile solvents (pull effect), and enhancement of drug solubility in the SC, which all promote the delivery of drugs to the hair follicles [38,39,40,41]. The relative extent of drug delivery greatly depends on the solubility of drugs and viscosity of individual permeation enhancer components. The concentration of volatile solvents (ethanol or isopropyl alcohol) used in the formulations can also generate the metamorphism phenomenon and affect drug delivery [38,42,43,44]. Thus, a supersaturated hydro-alcoholic vehicle system with an effective permeation enhancer may be the best approach for optimum drug delivery to the deep subcutaneous layers of the applied area.

The main objective of our present study was to design a vehicle system for topical AA that can immediately deliver drugs to hair follicles through the scalp of alopecia patients. To fulfill this aim, a solvent mixture system composed of volatile solvents and non-volatile co-solvents, such as permeation enhancers and hydrophilic agents, was developed to have enhanced solubility, skin permeability, and drug partitioning. The ideal composition of a topical formulation of AA (AA–TF) was identified based on drug permeation across an artificial membrane, as well as drug deposition and flux across human full-thickness skin. The optimum formulation (AA–TF) was further evaluated using in vitro cell proliferation assays with 5-(2,4-disulfophenyl)-3-(2-methoxy-4-nitrophenyl)-2-(4-nitrophenyl)-2H-tetrazolium (WST-8) and a scratch-wound recovery assay on keratinocyte (HaCaT) and human dermal papilla (HDP) cells. The stimulation of hair regrowth was examined in an androgenic mouse model treated with 1% minoxidil solution or commercially available oral finasteride. AA–TF was found to enhance the convergence of the telogen area, hair follicle length, weight of regrowth hair, and histology of hair follicles in the deep layer, as well as various hair-regrowth activating proteins, as determined by Western blot analysis.

## 2. Materials and Methods

### 2.1. Materials

Propylene glycol monocaprylate (type II) (Capryol 90; USP/NF grade, 98.4%) and diethylene glycol monoethyl ether (Transcutol P; EP/NF grade, 99.97%) were obtained from Gattefossé (Saint-Priest, France). Ethanol, isopropyl alcohol, benzyl alcohol, propylene glycol, polyethylene glycol 400 (PEG 400), octadic-9-enoic acid (oleic acid), isopropyl myristate, glycerin, decamethylcyclopentasiloxane (cyclomethicone), caprylic triglyceride, polyoxyethylene sorbitan monooleate 80 (Tween 80), and polyoxyethylene sorbitan monooleate 20 (Tween 20) were purchased from Sigma-Aldrich Inc. (St. Louis, MO, USA). All other solvents required for high-performance liquid chromatography (HPLC) and additional reagents for the experiments were purchased from Thermo Fisher Scientific Inc. (Waltham, MA, USA) and Sigma-Aldrich Inc.

### 2.2. Animals

Male C57BL/6J mice (6 weeks, 20 g) were purchased from G-Bio (Gwangju, Republic of Korea). The mice were housed in a maintained standard environment room at 25 ± 2 °C, 55 ± 10% relative humidity, and a 12-h light/dark cycle with free access to the standard laboratory diet (Nestlé Purina PetCare Company, St. Louis, MO, USA) and ion-sterilized tap water. Ethical approval was obtained from the Institutional Animal Care and Use Committee (IACUC) of Mokpo National University (Jeonnam, Republic of Korea; approval no. MNU-IACUC-2022-011). All animal experiments were performed in accordance with informed consent and ethical approval guidelines from the National Institutes of Health Guidelines for the Care and Use of Laboratory Animals and IACUC.

### 2.3. Preparation of AA from Stereocaulon japonicum

AA was prepared as described in the previous study [23]. Briefly, *Stereocaulon japonicum* collected from coastal rocks in southern Korea was provided by the Korean Lichen Research Institute (Sunchon National University, Sunchon-si, Republic of Korea). The dried thalli (146.68 g) were extracted with 1 L methanol at room temperature for 24 h using sonication. The extract was then filtered and dried in a rotary vacuum evaporator at 45 °C (Heidolph Instruments GmBH & CO, Schwabach, Germany). The crude extract (9.61 g) was dissolved in methanol and partitioned with water (50% hexane), before the addition of ethyl acetate. The water-soluble layer was separated using a separating funnel. Ethyl acetate was added to the water part and then it was further separated using a separating funnel containing butanol (1.88 g, gum). The butanol layer was subjected to open vacuum liquid chromatography using a C18 (50 × 260 mm) column with 20% and 100% methanol, to produce 10 fractions. Fraction 5 (76.6 mg) (water:methanol, 50:50, *v*/*v*) was further purified by reversed-phase HPLC equipped with a C18 column (10 × 250 mm) to obtain AA. The mobile phase was eluted at a flow rate of 3.0 mL/min with a time gradient of mobile phase B (0.5% [*v*/*v*] formic acid in acetonitrile) to mobile phase A (0.5% [*v*/*v*] formic acid in water): 0–5 min, 45% (*v*/*v*); 5–45 min, 45% (*v*/*v*); and 45–50 min, 100% (*v*/*v*).

### 2.4. Solubility of AA in Various Solvents

Drug solubility within a vehicle system is the most important factor when selecting volatile topical formulations, as it can enhance drug infiltration into deep skin layers. The excess solid method was used to examine the solubility of AA in various hydroalcoholic solvents (ethanol, isopropyl alcohol, benzyl alcohol, propylene glycol, and Transcutol P), lipophilic solvents (isopropyl myristate, caprylic triglyceride, oleic acid, glycerin, and cyclomethicone), and non-ionic surfactants (Capryol 90, PEG 400, Tween 20, and Tween 80) [45]. Briefly, 0.5 g of each solvent was vortex mixed with 10 mg of AA in a sealed glass container. Additional AA was continuously added to the mixture until a supersaturated solution was formed. The solutions were allowed to mix at 25 ± 1.0 °C in an isothermal shaker to reach equilibrium. After shaking for 24 h, the samples were centrifuged at 13,000 rpm for 15 min to remove the excess drug, and the supernatant was further diluted with a mixture of water and ethanol (50:50, *v*/*v*). The AA level in each solution was determined with an HPLC system (Waters Corp., Milford, MA, USA) connected with a Luna^®^ C18 column (4.6 × 250 mm, 5 µm, 100 Å, 20 µL sample injection). The flow rate of the mobile phase (0.5% formic acid in acetonitrile (*v*/*v*):0.5% formic acid in water (*v*/*v*), 50:50 (*v*/*v*)) was maintained at 1.0 mL/min and the AA concentration in each sample was quantified based on absorbance at 254 nm on a UV-VIS detector (model 2489; Waters Corp.) [23].

### 2.5. Preparation of AA–TFs

Ethanol was the primary volatile solvent used in the preparation of 1% (*w*/*w*) AA–TF (Table 1). Briefly, 10 mg of AA was weighed and dissolved in 300 mg of ethanol. Then, 99 mg (9.9%, *w*/*w*) of propylene glycol, Transcutol P, isopropyl myristate, PEG 400, or oleic acid were added to a glass vial containing AA in ethanol, which formed AA–TF#2, AA–TF#3, AA–TF#4, AA–TF#5, and AA–TF#6, respectively (Table 1). To evaluate skin hydration and minimize skin irritation induced by ethanol, 495 mg water (49.5%, *w*/*w*) was added to the AA–TF#1 solution, and 446 mg water (44.6%, *w*/*w*) was added to the AA–TF#2, AA–TF#3, AA–TF#4, AA–TF#5, or AA–TF#6 solutions. The combined effect of oleic acid and PEG 400 on AA skin infiltration was examined at 1:1, 1:2, and 1:3 weight ratios of oleic acid to PEG 400 for solutions containing AA–TF#7, AA–TF#8, and AA–TF#9. In addition, ethanol concentration was reduced to 33.0% (*w*/*w*) by adding propylene glycol (26.4%, *w*/*w*) and oleic acid (6.6%, *w*/*w*) in the formulation (i.e., AA–TF#9). Next, oleic acid (5 mg [0.5%, *w*/*w*], 10 mg [1.0%, *w*/*w*], 15 mg [1.5%, *w*/*w*], and 20 mg [2%, *w*/*w*]) was added to the formulations with a fixed amount of PEG 400 (297 mg [29.7%, *w*/*w*]) to assess the effect of oleic acid on drug permeation of the skin for AA–TF#10, AA–TF#11, AA–TF#12, and AA–TF#13. A fixed amount of ethanol (396 mg [39.6%, *w*/*w*] or 495 mg [49.5%, *w*/*w*]) was used to study how the weight ratio of oleic acid to water in the vehicle system affected drug partitioning on the skin using formulations AA–TF#14–#19. Finally, changes in drug content caused by precipitation or recrystallization in the vehicle system were monitored weekly for more than three months using an HPLC system with detection at 254 nm, as previously described.

### 2.6. In Vitro Permeability of AA–TF across an Artificial Membrane

A preliminary screen of the effects of individual components on the permeability of AA utilized an in vitro artificial skin permeability assay (Strat-M^®^; EMD Millipore, Temecula, CA, USA). The artificial membrane served as a barrier between the donor and receptor compartment of a Franz diffusion cell system with a diffusion area of 0.785 cm^2^ (Labfine, Gunpo-si, Republic of Korea). The receptor compartment was filled with 5 mL phosphate buffer saline (PBS, pH 7.4), which was assumed to maintain a sufficient sink condition, with a solubility of 1.10 ± 0.01 mg/mL. Indeed, the PBS solubility level was 11.0-fold higher than the maximum drug concentration (100 µg/mL) maintained in receptor compartments with 100% permeability. The receptor solution was stirred at 600 rpm using a magnetic stirrer and the membrane surface was maintained at 32 °C with a heating system during the experiments. After 30 min of equilibration, 50 µL of 1% (*w*/*w*) AA in ethanol or AA–TFs (all equivalent to 500 µg AA) was added to the donor chamber (0.785 cm^2^ permeation area). The receptor solution (500 µL) was collected at predetermined time points (0, 1, 2, 3, 4, 5, 6, 8, 10, and 24 h) and the equivalent volume of fresh PBS (pH 7.4) was used to refill the receptor compartment. The collected samples were filtered with a polyvinylidene fluoride (PVDF) membrane (0.45-µm pore size). The cumulative amount of AA that permeated the artificial membrane was determined using an HPLC system with 254 nm detection, as previously described.

### 2.7. In Vitro Infiltration and Deposition of AA in Full-Thickness Human Skin

Skin permeability and AA–TF flux was evaluated with an excised cadaveric full-thickness skin section (HansBiomed Corp., Daejeon, Republic of Korea). The skin was mounted between the donor and receptor chambers with the SC layer facing upwards and a 0.785 cm^2^ diffusion area. Each receptor compartment was filled with 5 mL of PBS (pH 7.4) and stirred continuously at 600 rpm to maintain a skin surface temperature of 32 °C. Before loading the sample solution, the diffusion cell system was allowed to equilibrate for >1 h to establish the integrity of the epidermis, which was confirmed by <10 g/m^2^/h transepidermal water loss (TEWL) and 1–2 kΩ of transepithelial electrical resistance (TEER). Then, 50 µL of 1% (*w*/*w*) AA in ethanol or AA–TFs (all equivalent to 500 µg AA) was added to the donor compartment with 0.785 cm^2^ of permeation area. The receptor solution (500 µL) was then withdrawn at various time points (0, 0.5, 1, 2, 3, 4, 5, 6, 8, 10, and 24 h) and replaced with the same volume of fresh PBS (pH 7.4). The withdrawn sample was filtered through a membrane filter (0.45 µm, PVDF), and AA that penetrated the sample was analyzed with HPLC analysis and 254-nm detection, as previously described.

Drug deposition was evaluated on different skin layers during equilibrium. The effectiveness of the once-a-day topical application of AA–TFs was analyzed at 24 h after drug loading by disassembling the diffusion cells and carefully isolating the skin before washing it five times with deionized water. Residual water was absorbed with tissue paper and the skin exposed to the drug solution was cut and weighed. The SC was isolated by tape-stripping the skin 35 times with D-Squame (CuDerm, Dallas, TX, USA) [46,47]. Then the epidermis and dermis were separated, and the remaining skin was immersed overnight in 5 mL of ice-cold dispase solution [0.5% (*w*/*v*)] [45]. The isolated SC, epidermis, and dermis layers were cut into small pieces and the drug retained in each layer was extracted with ethanol (2 mL). After drying the extracted solution with a centrifugal evaporator (Genevac Ltd., Ipswich, UK), the residue was reconstituted with 200 µL of ethanol. The final solution was filtered through a PVDF membrane (0.45-µm pore size) and the AA quantity in each sample was determined with 254 nm detection and HPLC analysis, as previously described.

### 2.8. In Vitro Cell Proliferation Assay

The biological activity of AA–TF was compared to minoxidil using cell proliferation assays carried out with HaCaT and HDP cells (PromoCell GmbH, Heidelberg, Germany). Briefly, HaCaT or HDP cells were seeded onto 96-well plates at a density of 2.5 × 10^3^ cells/well in 100 µL of Dulbecco’s modified Eagle’s medium (DMEM) containing either 10% fetal bovine serum (FBS; *v*/*v*) with 1% penicillin/streptomycin (*v*/*v*) or HDP medium containing 4% fetal calf serum (FCS) (*v*/*v*) or 4% bovine pituitary extract (*v*/*v*) in 1 µg/L recombinant human fibroblast growth factor and recombinant human insulin (5 mg/L) (PromoCell GmbH, Heidelberg, Germany). After a 24 h incubation at 37 °C, the HaCaT and HDP cells were further cultured in serum-free DMEM or HDP medium for another 24 h. The cells were then treated with serially diluted AA–TF#1, AA–TF#15, and minoxidil in ethanol at a concentration of 1, 5, 10, 50, and 100 µM drug in each vehicle solution. After incubation for an additional 24 h, cell viability was measured by adding 100 μL of WST-1 reagent (Roche Diagnostics GmbH, Mannheim, Germany) dilution with DMEM or HDP medium (10%, *v*/*v*) to each well. After a 4 h incubation, the absorbance at 450 nm was measured using a multimode microplate reader (PerkinElmer, Waltham, MA, USA). The percentage of viable cells in the treatment group was calculated by comparing the values to those from untreated cells.

### 2.9. In Vitro Scratch-Wound Recovery Assay

In vitro scratch-wound recovery assays were performed to investigate the promotion of cell proliferation by AA–TF, as well as its migration. Briefly, HaCaT or HDP cells were seeded on each well of a collagen-coated 96-well plate (ImageLock; Essen BioScience, Inc., Ann Arbor, MI, USA) at a density of 1 × 10^4^ cells in 100 µL of DMEM containing 10% FBS (*v*/*v*) and 1% penicillin/streptomycin (*v*/*v*), or 2.5 × 10^4^ cells in 100 µL of HDP medium containing 4% FCS (*v*/*v*), 4% bovine pituitary extract (*v*/*v*), 1 µg/L recombinant human fibroblast growth factor, and recombinant human insulin (5 mg/L), respectively. After incubating the cells at 37 °C for 48 h to produce confluent monolayers, cell-free scratches with a width of 700–800 µm were created using a 96-pin IncuCyte wound-maker (Essen BioScience, Inc.) and the detached cells were washed three times with PBS (pH 7.4). The HaCaT or HDP cells were further treated with 100 µL of the vehicle of AA–TF#1, the vehicle of AA–TF#15, AA–TF#1, AA–TF#15, or minoxidil in ethanol diluted with DMEM containing 0.5% FBS (50 µM AA equivalent) or HDP medium containing 0.5% FCS (10 µM AA equivalent), respectively. The plates containing the HaCaT or HDP cells were incubated at 37 °C in an IncuCyte FLR microscope (Essen BioScience, Inc.) for 24 h, and wound recovery was quantified using IncuCyte FLR image analysis software (Essen BioScience, Inc.), which detected cell edges and generated an overlay mask to calculate relative wound density.

### 2.10. Efficacy of In Vivo Hair Regrowth

The ability of AA–TF to regrow hair was evaluated for androgenic alopecia, where the dorsal hair of each mouse was removed using an electric animal clipper and duct tape. The mice were randomly divided into six groups (8 mice/group). All mice, except the control group, were topically treated with 100 µL of 0.5% testosterone in ethanol once daily for 18 days to induce androgenic alopecia [10]. One hour after treatment with 0.5% testosterone in ethanol, each group was treated with either the control (androgenic alopecia, once-daily topical treatment with AA–TF#15 vehicle), minoxidil (1%, once-daily topical treatment with 1% minoxidil in vehicle composed of ethanol, propylene glycol, and water [60:20:20, *v*/*v*/*v*]), AA in EtOH (1%, once-daily topical treatment with 1% AA in ethanol), AA–TF#15 (1%, once-daily topical treatment with AA–TF#15 containing 1% AA), and finasteride (once-daily oral administration of finasteride [1 mg/kg]). Each sample (25 µL) was applied to the dorsal skin or orally administered (100 µL finasteride dispersed in water) once per day for 18 days. Hair regrowth on the dorsal skin was examined using photographs from 0, 3, 6, 9, 12, 15, and 18 days after treatment. The rate of hair growth was estimated by determining the areas of telogens (pink skin), areas of conversion from telogens to anagens (black skin), and areas covered with hair using ImageJ software (NIH, Bethesda, MD, USA). Regrown hair from the mice was cut and weighed using an analytical balance and hair follicle length was measured 18 days after treatment. Dorsal skin tissues were excised and immediately fixed in 4% neutral phosphate-buffered formalin (*v*/*v*). The skin tissues were then embedded in paraffin blocks, cut to obtain both longitudinal and transverse sections with a thickness of 5 μm, and stained with hematoxylin and eosin (H&E) to assess gross histopathological changes, as well as hair follicles in the treatment areas.

### 2.11. Western Blot Assay

Protein levels were monitored in the skin by homogenizing isolated skin tissue in cold lysis buffer at 3650 rpm, 6.00 m/s linear speed, and 6 cycles for 30 s (Bioprep-24R, Allsheng, Hangzhou, China). The samples were centrifuged at 13,500 rpm for 10 min at 4 °C. Protein concentrations in the lysate were determined by BCA assay (Thermo Fisher Scientific Inc.) and the extracted protein was separated by SDS-PAGE. Antibodies against aldehyde dehydrogenase 1 (ALDH1) (Santa Cruz Biotechnology, Dallas, TX, USA), α-tubulin, β-catenin, pyruvate kinase M2 (PKM2, Cell Signaling Technology, Danvers, MA, USA), and cyclin D1 (Merck, Kenilworth, NJ, USA) were detected using horseradish peroxidase (HRP)-conjugated secondary antibody (Thermo Fisher Scientific Inc.) and the Immobilon Western Chemiluminescent HRP Substrate Kit (Millipore, Billerica, MA, USA). The density of the band was measured to obtain the relative density using loading controls. Values are expressed as arbitrary densitometric units that correspond to signal intensity.

### 2.12. Statistical Analysis

All data are expressed as mean ± standard deviation (SD) or standard error of the mean (SEM). A *p*-value less than 0.05 was considered statistically significant. One-way analysis of variance (ANOVA) followed by Tukey’s multiple comparison test was performed for the analysis of three or more mean values.

## 3. Results

### 3.1. Solubility of AA

**Figure 1:**
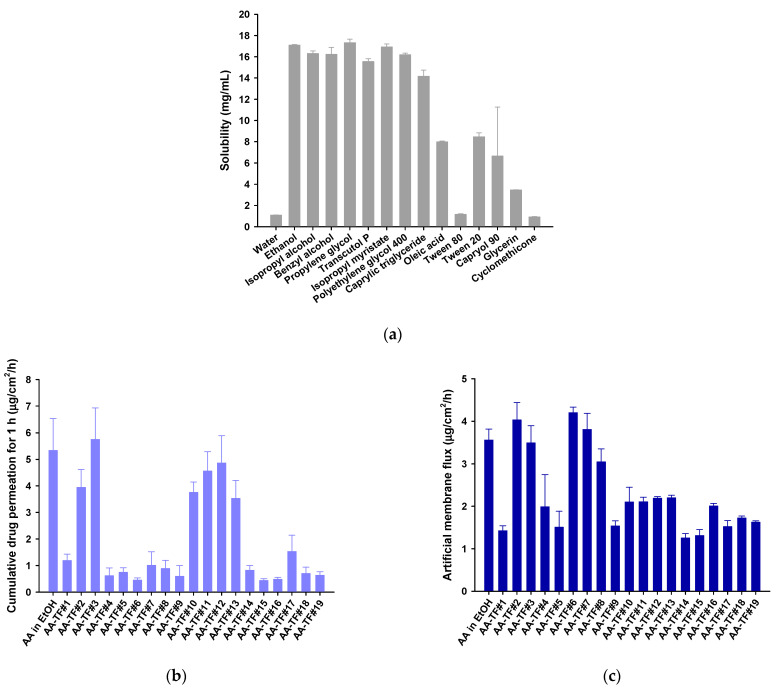
(**a**) Solubility of atraric acid (AA) in various solvents and surfactants. In vitro infiltration of AA using topical formulations (AA–TFs) into an artificial membrane; (**b**) Cumulative drug permeation for the initial first hour; and (**c**) Flux of AA in ethanol or AA–TFs. Values are presented as mean ± SDs (*n* = 4 for each group).

A vehicle system containing suitable solvents capable of solubilizing the drug, as well as in the applied area, is a key factor that determines the efficacy of a topical dosage. Therefore, the solubility of AA in various excipients, including water, hydroalcoholic as well as lipophilic solvents, and non-ionic surfactants, was analyzed in this study (Figure 1a). The solubility of AA in water was only 1.09 ± 0.01 µg/mL, indicating it is less soluble in the aqueous phase. The solubility of AA in ethanol, isopropyl alcohol, benzyl alcohol, propylene glycol, Transcutol P, glycerin, and PEG 400 was drastically elevated when compared to AA in water. Among these hydroalcoholic solvents, ethanol and propylene glycol demonstrated maximum solubility, which was 17.1 ± 0.05 and 17.3 ± 0.33 µg/mL, respectively. The lipophilic solvents oleic acid, isopropyl myristate, and caprylic triglyceride increased the solubility of AA by 7.32-, 15.5-, and 13.0-fold compared to AA in water, respectively. However, non-ionic surfactants, such as Tween 80, Tween 20, and Capryol 90, as well as a spreading agent, cyclomethicone, reduced the solubility of AA compared to hydroalcoholic and lipophilic solvents. Therefore, ethanol was used as a vehicle solvent, while other surfactants, such as propylene glycol, Transcutol P, isopropyl myristate, PEG 400, and oleic acid were included in screens and the design of an effective vehicle system for AA.

### 3.2. Design and Analysis of Artificial Membrane Permeability of AA–TF

An artificial skin permeability study was performed using the Franz diffusion cell system to identify effective topical formulations for AA permeation. Based on the high solubility and enhanced permeability in ethanol, AA–TF was prepared by dissolving it in 100% ethanol. Although AA had enhanced permeability across the artificial membrane in ethanol (5.34 µg/cm^2^/h for the first hour; Figure 1b), the high concentration of ethanol (99.0%) could cause skin irritation, including redness, burning sensations, erythema, and itching, as well as drastically disturbing the skin barrier function [48,49]. Therefore, 49.5% was selected as a minimum concentration of ethanol to maintain the solubilized form of AA and AA–TF#1. AA–TF#1 displayed a permeation value of 1.19 ± 0.244 µg/cm^2^/h for 1 h with a flux rate of 1.43 ± 0.117 µg/cm^2^/h (Figure 1b,c).

AA–TF#2 and AA–TF#3 demonstrated maximum permeation of AA during the first hour after treatment, which was 231% and 383% higher than that of AA–TF#1, respectively. However, the flux value of AA–TF#6 exceeded that of AA–TF#2–5, with a 194% greater value compared to AA–TF#1 (Figure 1b,c). This was likely to have resulted from the ability of oleic acid to maintain the efficient partitioning of drugs in the membrane, compared to other surfactants. We then examined the effects of combined oleic acid and PEG 400 on the permeability of AA in AA–TF#7–15. An increasing reduction in permeability and flux was observed with AA–TF#7, AA–TF8, and AA–TF#9, in that order, which can be correlated with reduced ethanol content and increased PEG 400 concentration. Ethanol was therefore fixed at 49.5% in the formulation and additional combinations of PEG 400 (29.7%) and oleic acid (0.5, 1.0, 1.5, and 2.0%) were screened using AA–TF#10, AA–TF#11, AA–TF#12, and AA–TF#13, respectively.

The permeation of AA was enhanced in AA–TF#10–13 compared to AA–TF#7–9 during the initial first hour, revealing the important role of ethanol in the initial phase of drug permeation (Figure 1b,c). AA–TF#12 demonstrated maximum drug permeation during the first hour, with values 29.6%, 6.64%, and 37.8% higher than those of AA–TF#10, AA–TF#11, and AA–TF#13, respectively. However, the lower flux of AA–TF#10–13 compared to AA–TF#7–9 is likely to be related to the lower concentration of oleic acid (Figure 1b,c). Therefore, AA–TF#14–19 was selected for a screen to assess the permeation-enhancing effect of oleic acid at different percentages (5%, 9.9%, and 19.8%). The permeation of AA during the first hour after AA–TF#14–19 treatment was lower than that of AA–TF#10–13 and AA–TF#2–3 (Figure 1b,c). The flux observed with AA–TF#14–16 and AA–TF#17–18 increased as the concentration of oleic acid increased. Based on the initial partitioning and flux of the drug during 24 h of treatment, several AA–TFs were selected for in vitro human skin permeability tests.

### 3.3. In Vitro Infiltration and Deposition of AA in Full-Thickness Human Skin

**Figure 2:**
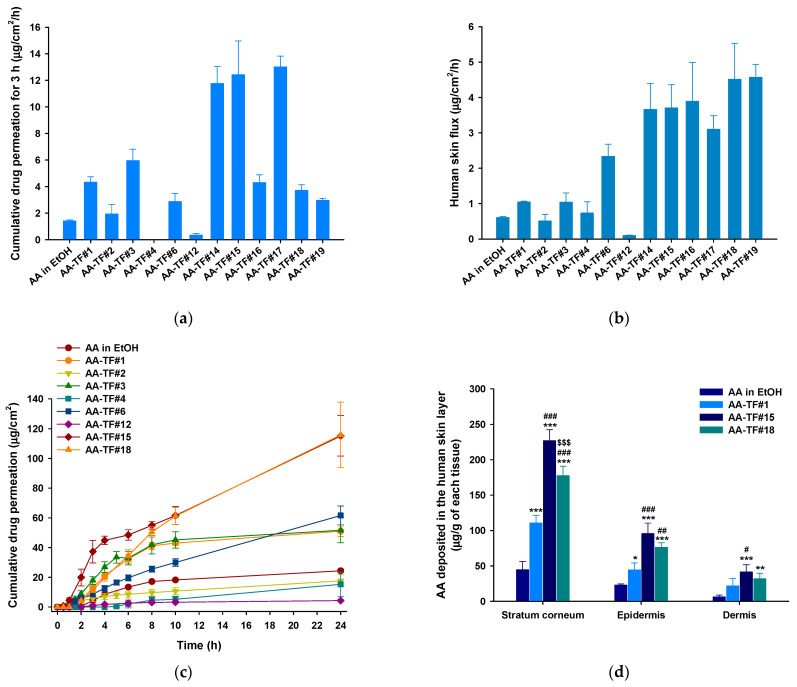
In vitro full-thickness human skin permeability of atraric acid (AA) in ethanol (EtOH) and topical formulations (AA–TFs): (**a**) Cumulative drug infiltration in human skin during the initial 3 h after treatment; (**b**) Skin flux with 1% AA in EtOH or AA–TFs over 24 h; (**c**) Time-course curve of cumulative AA permeation infiltration of 1% AA in EtOH or AA–TFs for 24 h; (**d**) AA deposited in the human stratum corneum, epidermis, and dermis using 1% AA in EtOH, AA–TF#1, AA–TF#15, and AA–TF#18 24 h after loading. * *p* < 0.05, ** *p* < 0.01, and *** *p* < 0.001 compared to AA in EtOH; ^#^ *p* < 0.05, ^##^*p* < 0.01, ^###^ *p* < 0.001 compared to AA–TF#1; ^$$$^ *p* < 0.001 compared to AA–TF#15. Values are presented as means ± SDs (*n* = 4 for each group).

The in vitro permeation of AA in ethanol and AA–TFs across human skin was evaluated based on the infiltration of the drug after 3 h, skin flux, and depositions of the drug in the different skin layers (Figure 2). The drug permeation of AA–TF#1 was 205% higher during the first 3 h, and skin flux was 1.71-fold greater than AA in ethanol. Three hours after drug loading, cumulative permeated AA was 36.8% higher for AA–TF#2, compared to AA in ethanol (Figure 2a,b). Skin permeation of AA from AA–TF#2, AA–TF#4, and AA–TF#6 was not significantly higher than that of AA–TF#1 (Figure 2a,b). Among the AA–TFs that contained additional surfactant, AA–TF#3 showed the largest increase (37.7%) in cumulative AA permeation during the initial 3 h, as compared to AA–TF#1. Analysis of PEG 400 and oleic acid combinations indicated that AA–TF#12 had reduced cumulative permeation of AA after 3 h by 1133% and 1598%, and reduced skin flux by 987% and 984% compared to AA–TF#1 and AA–TF#3, respectively (Figure 2a,b). Based on the flux trends observed with AA–TF#14–16 and AA–TF#17–19, the rate of drug permeation was found to be dependent on the concentration of oleic acid. Cumulative drug infiltration levels of AA–TF#14, AA–TF#15, and AA–TF#17 during the initial three hours were enhanced by 97.2%, 108%, and 118%, respectively, compared to AA–TF#3, which previously displayed maximum permeation. These results indicate that the ratio of ethanol, oleic acid, and water is important for determining drug partitioning in the skin during the initial phase.

Infiltration was compared after 24 h and indicated AA–TF#1 had a cumulative drug permeation of 51.1 ± 3.41 µg/cm^2^, which was 109% higher than that of AA in ethanol (Figure 2b,c). Compared to AA–TF#1–4, AA–TF#6 exhibited significantly more drug infiltration at 24 h, with 20.7%, 249%, 19.3%, and 302% higher values than those from AA–TF#1, AA–TF#2, AA–TF#3, and AA–TF#4, respectively (Figure 2b,c). The analysis of AA–TF#6 indicates oleic acid effectively promoted drug permeation, as compared to propylene glycol, Transcutol P, and isopropyl myristate. However, the lower partitioning of AA observed with AA–TF#12 indicated a 1331% and 466% reduction during the first 24 h compared to AA–TF#6 and AA in ethanol, respectively. These results indicate that combining PEG 400 with oleic acid cannot efficiently promote the infiltration of AA across human skin. However, the permeation of AA from AA–TF#15 was 86.8% higher and 87.8% for AA–TF#18, as compared to AA–TF#6 after 24 h. These findings may result from synergistic variations in oleic acid, ethanol, and water combinations in the AA–TF solutions during drug partitioning (Figure 2b,c).

AA deposition in various skin layers was analyzed after the application of AA–TF#15 and AA–TF#18 for 24 h, compared to AA in ethanol and AA–TF#1 (Figure 2d). Drug deposition was the highest in the SC, followed by the epidermis, and finally, the dermis. The deposition of AA from AA–TF#15 was 408%, 104%, and 27.9% higher in the SC layer, 319%, 115%, and 25.3% greater in the epidermis, and 570%, 90.9%, and 30.2% higher compared to the respective layers treated with AA in ethanol, AA–TF#1, and AA–TF#18, respectively (Figure 2d).

Based on the overall results of the human skin infiltration (initial drug permeation and flux) and deposition studies, AA–TF#15 was found to be an optimized formulation, and additional experiments were conducted with this formulation in comparison to AA–TF#1 (i.e., 1% AA in 50% ethanol) or 1% AA in ethanol.

### 3.4. Cell Proliferation Assay

**Figure 3:**
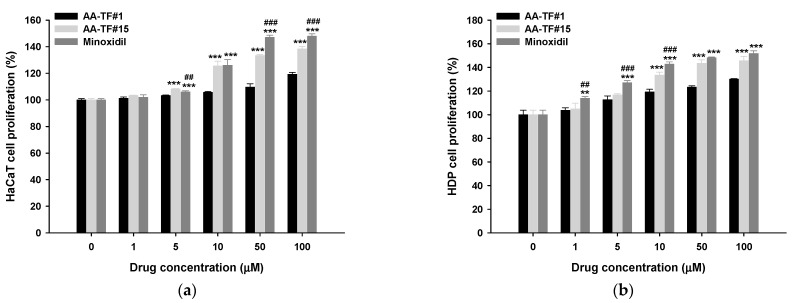
Relative cell proliferation of (**a**) keratinocytes (HaCaT) and (**b**) human follicle dermal papilla (HDP) cells after treatment with atraric acid (AA) topical formulations (AA–TF#1 and AA–TF#15) or minoxidil in ethanol (EtOH). Solutions were diluted to drug concentrations of 0, 1, 5, 10, 50, and 100 µM. ** *p* < 0.01, *** *p* < 0.001 compared to AA–TF#1; ^##^ *p* < 0.01, ^###^ *p* < 0.001 compared to AA–TF#15 at the same drug concentration. Values are represented at mean ± SD (*n* = 4 for each group).

The biological effect of AA was examined after it was incorporated into AA–TF by performing a cell proliferation assay. HaCaT and HDP cells were treated with AA–TF#1, AA–TF#15, or minoxidil (Figure 3). The effect of AA on cell proliferation was dose-dependent between 1 and 50 µM. Cell proliferation was elevated in HaCaT by 19.2% 24 h after treatment with 100 µM of AA–TF#1. However, cell proliferation was not significantly enhanced by increasing concentrations of AA (50 µM to 100 µM), which may have been due to saturation of the receptor-mediated effect, as well as elevated levels of ethanol in the treatment medium containing higher drug concentrations. When HaCaT cells were treated with AA–TF#15 (50 µM AA equivalent) for 24 h, cell proliferation value increased by 133%, which was 23.7% greater than AA–TF#1 (Figure 3a). Minoxidil (50 µM) induced 46.9% more cell proliferation in HaCaT cells, which is only 6.72% higher than proliferation induced by AA–TF#15. These results indicate that AA–TF#15 has about the same cellular proliferation activity as minoxidil at the same molar concentration.

Cellular proliferation in HDP cells was similar to that of HaCaT cells (Figure 3b). Treatment of HDP cells with AA–TF#1 (50 µM AA equivalent) for 24 h induced 23.3% more cell proliferation than untreated cells (Figure 3b). However, a 24 h incubation with 50 µM of AA–TF#15 increased cellular proliferation by 143%, which is 20.0% higher than AA–TF#1. On the other hand, no significant difference was observed with minoxidil (50 µM).

### 3.5. In Vitro Scratch-Wound Recovery Assay

**Figure 4:**
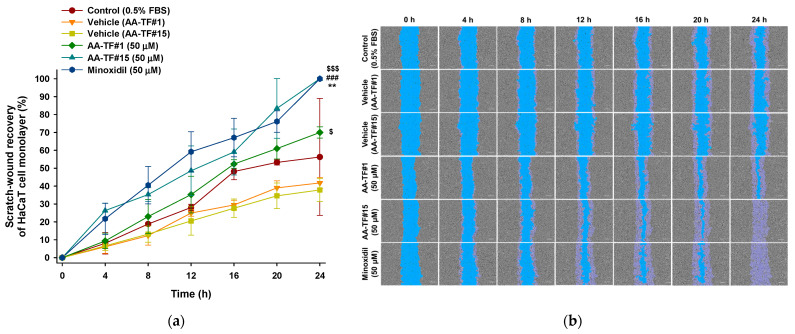
In vitro assay for determination of relative scratch-wound recovery of HaCaT cells incubated with 1% atraric acid (AA) topical formulations (AA–TF#1 and AA–TF#15), 1% minoxidil in ethanol (EtOH), or vehicles of AA–TF#1 or #15 diluted to a drug concentration equivalent to 50 µM: (**a**) Time-course curve of relative scratch-wound recovery area of HaCaT cell monolayers. ** *p* < 0.01 compared to control (0.5% FBS); ^###^ *p* < 0.001 compared to vehicle (AA–TF#1); ^$^ *p* < 0.05, ^$$$^ *p* < 0.001 compared to vehicle (AA–TF#15). Values represent mean ± SD (*n* = 4); (**b**) Representative microscope images of scratch-wounds of HaCaT cells. Scale bar = 200 µm.

**Figure 5:**
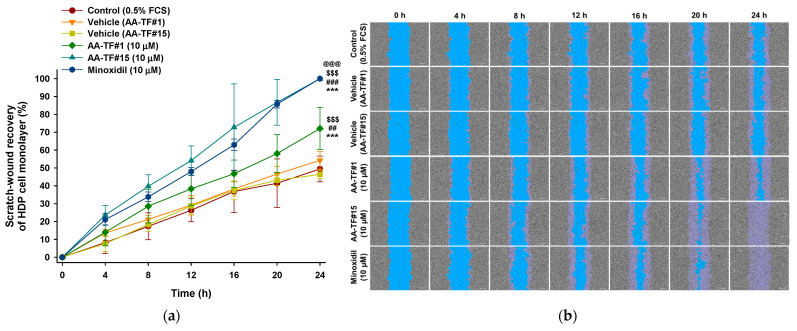
In vitro scratch-wound healing in HDP cells following treatment with 1% atraric acid (AA) topical formulations (AA–TF#1 and AA–TF#15), 1% minoxidil in ethanol (EtOH), or vehicles of AA–TF#1 or #15 diluted to a drug concentration equivalent to 10 µM: (**a**) Time-course showing relative scratch-wound recovery of HDP cell monolayer. *** *p* < 0.001 compared to control (0.5% FCS); ^##^ *p* < 0.01, ^###^ *p* < 0.001 compared to vehicle (AA–TF#1); ^$$$^ *p* < 0.001 compared to vehicle (AA–TF#15); ^@@@^ *p* < 0.001 compared to AA–TF#1 (10 µM). Values represent the mean ± SD (*n* = 4); (**b**) Representative microscope images of HDP cell scratch-wounds. Scale bar = 200 µm.

The therapeutic potential of AA, as well as the cellular proliferation and migration activities, were assessed using an in vitro wound healing assay. Confluent HaCaT and HDP cells were scratched and compared with non-treated and minoxidil-treated groups. The initial wound area was marked as a reference for the quantification of wound recovery. The recovery rate was calculated after the 24 h treatment using AA–TF#1, AA–TF#15, and minoxidil. Simultaneous treatment with AA–TF#1 vehicle (with 0.5% ethanol) and AA–TF#15 vehicle (containing 0.2% ethanol) for 24 h did not indicate any interference in cell migration in either HaCaT or HDP cells. These results indicate that the dilution factor of samples is appropriate for the study, and they do not cause cytotoxic effects (Figure 4 and Figure 5).

AA–TF#1 demonstrated 35.2% and 38.3% wound closure after 12 h of treatment with the equivalent of 50 µM AA for HaCaT cells and 10 µM for HDP cells, respectively. Minoxidil treatment, which is the positive control, recovered 59.2% and 47.9% of scratched wounds in HaCaT and HDP cells after 12 h at an equivalent of 50 µM and 10 µM drug, respectively. On the other hand, the overall wound closure rate in HaCaT as well as HDP cells was higher in the AA–TF#15 treatment, compared to that in AA–TF#1. After a 12 h incubation, AA–TF#15 showed elevated scratch-wound closure at 48.6% in HaCaT and 54.1% in HDP cells, respectively. Moreover, wound closure was significantly accelerated with AA–TF#15 treatment after 20 h, and larger wound closure areas were maintained in both cell types after treatment, as compared to minoxidil. AA–TF#15 treatment produced 100% wound closure in HaCaT and HDP cells after a 24-h incubation, such as minoxidil. Based on the enhanced skin permeability and cell proliferation activity, AA–TF#15 was selected for the in vivo studies.

### 3.6. In Vivo Hair Regrowth Study

Hair growth was evaluated in alopecia mice after application with AA–TF#15. Dorsal skin areas were characterized as appearing black (anagen) due to hair regrowth or pink (telogen) due to the lack of hair regrowth. Growth was measured on days 0, 3, 6, 9, 12, 15, and 18 of treatment. The overall conversion of telogen to anagen areas, as well as regrowth of the hair in the androgenic alopecia control, was significantly lower compared to the normal control. The treatment group containing AA–TF#15 (1%), minoxidil (1%), and finasteride (oral), displayed an enhanced rate of telogen to anagen phase conversion, as well as hair regrowth when compared to both controls (normal and androgenic alopecia). No variations in the skin of mice from each treatment group were observed until the third day of treatment.

**Figure 6:**
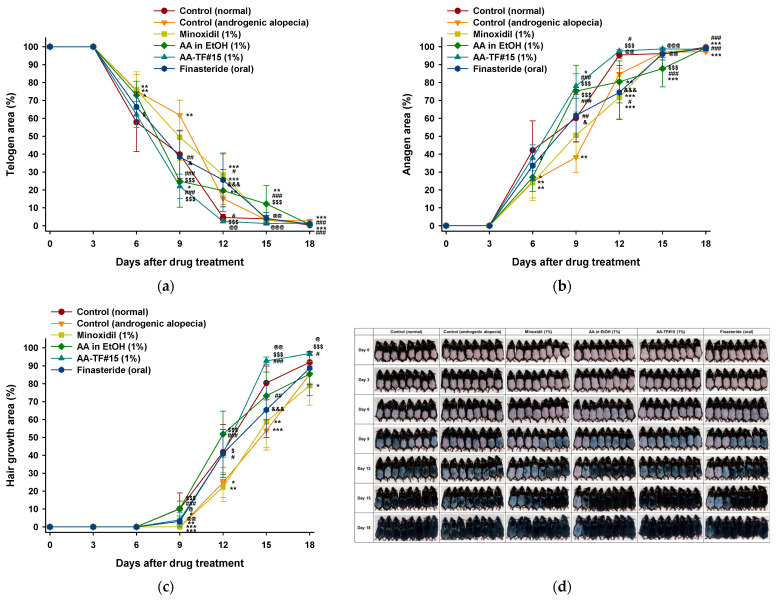
Efficacy of in vivo hair regrowth using once-daily topical treatment of 1% atraric acid (AA) in ethanol (EtOH), AA–TF#15, and 1% minoxidil topical solution, or once-a-day oral administration of finasteride (10 mg/kg) in androgenic alopecia mice: (**a**) Time-course curve of the dorsal skin area showing a pink color (telogen); (**b**) Time-course curve of changes in dorsal skin color from pink to black, indicating hair regrowth (anagen); (**c**) Time-course curve of hair growth on dorsal skin. * *p* < 0.05, ** *p* < 0.01, *** *p* < 0.001 compared to control (normal); ^#^ *p* < 0.05, ^##^ *p* < 0.01, ^###^ *p* < 0.001 compared to control (androgenic alopecia); ^$^ *p* < 0.05, ^$$$^ *p* < 0.001, compared to minoxidil (1%); ^@^ *p* < 0.05, ^@@^ *p* < 0.01, ^@@@^ *p* < 0.001 compared to AA in EtOH (1%); ^&^ *p* < 0.05, ^&&&^ *p* < 0.001 compared to AA–TF#15 (1%). Values are presented as mean ± SEM (*n* = 8 for each group); (**d**) Representative photographs of mouse dorsal skin showing hair regrowth at 0, 3, 6, 9, 12, 15, and 18 days after treatment.

A change in the telogen to anagen phase in treated mice was observed on day 6 of treatment (Figure 6a,b). Mice treated with 1% minoxidil and 1% AA in ethanol did not show changes in telogen-to-anagen area conversions, as compared to the control (androgenic alopecia) (Figure 6a,b). However, AA–TF#15 (1%) and finasteride (oral) demonstrated a 33.7% and 33.5% conversion, respectively, of telogen-to-anagen phase areas on day 6 (Figure 6a,b). Moreover, the latency associated with the color change of the treated area from pink to black indicated an early anagen phase. The average latency for all mice in each group was also calculated. The latency of AA–TF#15 (1%) was 5.80 ± 1.58 days, which was 29.4%, 32.8%, 24.2%, 10.3%, and 5.22% shorter than those found in the normal control, androgenic alopecia control, minoxidil (1%), AA in ethanol (1%), and finasteride (oral) groups, respectively.

After 9 days of treatment, AA in ethanol (1%) and AA–TF#15 (1%) demonstrated a 75.1% and 78.0% conversion of telogen-to-anagen area, which was the highest rate among all treatment groups (Figure 6a,b). Hair regrowth was also observed in mice treated with the control (normal), AA in ethanol (1%), AA–TF#15 (1%), and finasteride (oral), which represented 10.0%, 10.2%, 3.95%, and 3.04% of the hair regrowth area, respectively (Figure 6c,d). After twelve days of treatment, 97.58 ± 2.28% of dorsal skin areas displayed signs of anagen in the AA–TF#15 (1%)-treated mice (Figure 6b). The anagen skin areas were 12.8%, 25.9%, and 23.1% larger than those in the control (androgenic alopecia), minoxidil (1%), and finasteride (oral)-treated mice, respectively. The hair regrowth area was greatest in mice treated with AA in ethanol (1%, *w*/*w*) (51.5% hair regrowth) after twelve days, which was 10.0%, 10.9%, 29.5%, and 10.8% more than the control (normal), AA–TF#15 (1%), minoxidil (1%), and finasteride (oral) treated mice, respectively (Figure 6c,d).

On day 15, the area covered by regrown hair was significantly greater in the AA–TF#15 (1%)-treated mice, which was 19.5%, 34.0%, and 27.4% greater than in those treated with AA in ethanol (1%), minoxidil (1%), and finasteride (oral), respectively (Figure 6c,d). Overall hair regrowth in mice treated with AA–TF#15 (1%) was the highest among all treatment groups at 96.7 ± 2.57% on day 18.

### 3.7. Hair Weight and Histological Evaluation

**Figure 7:**
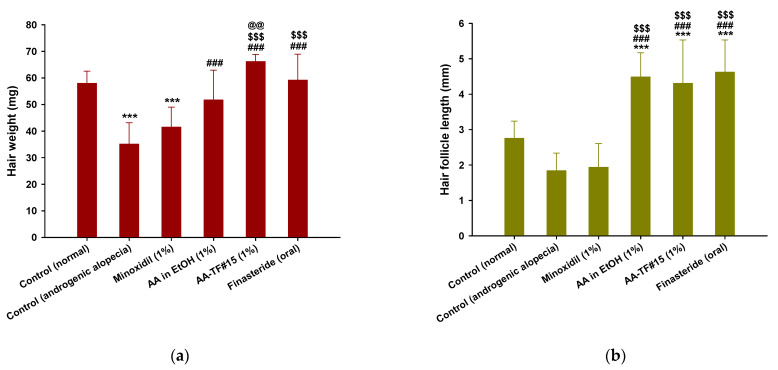

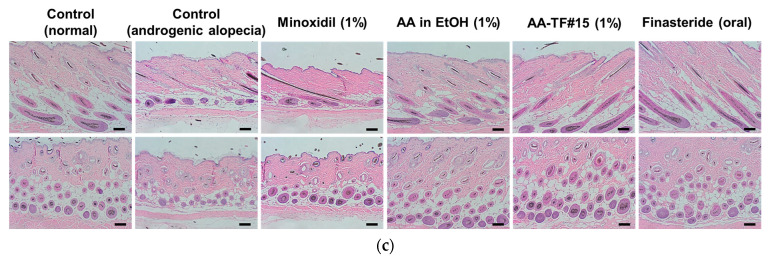
(**a**) Hair weight from each group at 18 days after treatment; (**b**) Hair follicle length from each group after 18 days of treatment. *** *p* < 0.001 compared to control (normal); ^###^ *p* < 0.001 compared to control (androgenic alopecia); ^$$$^ *p* < 0.001 compared to minoxidil (1%); ^@@^ *p* < 0.01 compared to AA in EtOH (1%). Values are presented as mean ± SD (*n* = 8 for each group); (**c**) Representative light micrographs of longitudinal and transverse sections of androgenic alopecia skin stained with hematoxylin and eosin (H&E) for quantitation of follicle number and diameter. Scale bar = 100 µm.

Newly grown hairs on the dorsal skin of the test groups were weighed at the end of treatment. The weight of hair from the control (androgenic alopecia) mice was less than that of the control (normal), which was likely to have been caused by delayed hair regrowth due to androgenic alopecia (Figure 7a). The hair weights of mice treated with AA in ethanol (1%), minoxidil (1%), and finasteride (oral) were 47.2%, 18.1%, and 68.5% higher than that of the control (androgenic alopecia), respectively. Androgenic hair regrowth observed with AA–TF#15 (1%) treatment was superior to other treatment groups 18 days after treatment, including the control (normal). The hair weight from AA–TF#15 was 27.9%, 59.4%, and 11.7% higher than for AA in ethanol (1%), minoxidil (1%), and finasteride (1 mg/kg orally), respectively, which suggests the formation of dense hair follicles after treatment with AA–TF#15 (1%) (Figure 7a).

Hair follicle length was measured using randomly plucked and newly grown hairs from the dorsal skin on the last day of treatment. As shown in Figure 7b, hair follicles from mice treated with AA–TF#15 (1%), AA in ethanol (1%), and finasteride (oral) were longer than those of the androgenic alopecia and normal control. Hair follicles from the normal control were 1.50-fold longer than those of the androgenic alopecia control, which confirms the importance of androgens in hair follicle development. Eighteen days after treatment, follicles from AA–TF#15 (1%), AA in ethanol (1%), and finasteride (oral) treated mice were found to be 2.34-, 2.43-, and 2.51-fold longer than those of the control (androgenic alopecia), and 2.22-, 2.30-, and 2.38-fold longer than those of the minoxidil (1%)-treated mice, respectively (Figure 7b).

Enhanced hair regrowth in androgenic alopecia mice treated with AA–TF#15 was confirmed by analyzing hair follicle density in the treatment area using histological H&E staining of both the longitudinal and transverse dimensions on the final day of treatment (Figure 7c). After 18 days, the dorsal skin treated with AA–TF#15 (1%), AA in ethanol (1%), and minoxidil (1%) did not exhibit any signs of irritation or inflammation, such as epidermal thickening or infiltration of inflammatory cells. The topical application of AA–TF#15 substantially increased the number of hair follicles compared to the normal control, androgenic alopecia control, AA in ethanol (1%), minoxidil (1%), and finasteride (oral) groups. The number and diameter of hair follicles on day 18 were significantly elevated in AA–TF#15 (1%)-treated mice compared to all other groups, including the 1% minoxidil topical solution and oral finasteride-treated mice (Figure 7c).

### 3.8. Effects of AA–TF on Activation of the Wnt/β-Catenin Pathway in Androgenic Mice

**Figure 8:**
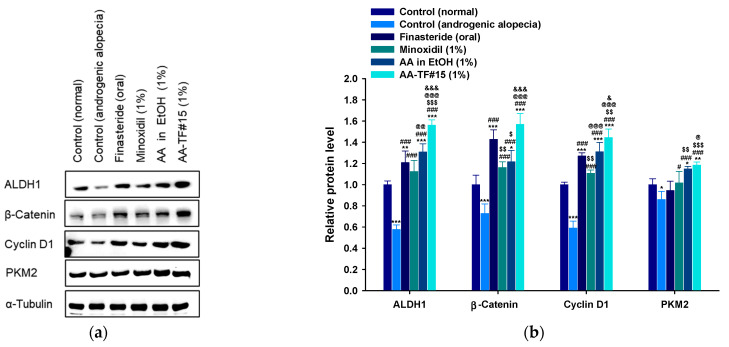
Expression of ALDH-1, β-catenin, cyclin D1, and PKM2 proteins in the dorsal skin of mice at 18 days after treatment: (**a**) Western blot analyses of ALDH-1, β-catenin, cyclin D1, and PKM2 proteins; (**b**) Relative protein levels of ALDH-1, β-catenin, cyclin D1, and PKM2 in the skin using α-tubulin and actin as loading controls. * *p* < 0.05, ** *p* < 0.01, *** *p* < 0.001 compared to control (normal); ^#^ *p* < 0.05, ^###^ *p* < 0.001 compared to control (androgenic alopecia); ^$^ *p* < 0.05, ^$$^ *p* < 0.01, ^$$$^ *p* < 0.001 compared to orally administered finasteride; ^@^ *p* < 0.05, ^@@^ *p* < 0.01, ^@@@^ *p* < 0.001 compared to minoxidil (1%); ^&^ *p* < 0.05, ^&&&^ *p* < 0.001 compared to AA in EtOH (1%). Values are presented as mean ± SD (*n* = 4 for each group).

The biological response of AA was further examined in mouse skins treated with AA in ethanol (1%) and AA–TF#15 (1%). Proteins were examined and compared to expression levels in the skins of normal and androgenic alopecia controls, as well as from mouse skins treated with finasteride (oral) and a minoxidil solution (1%). The level of ALDH1, β-catenin, cyclin D1, and PKM2 decreased by 73.6%, 37.4%, 69.3%, and 16.4%, respectively, in the androgenic alopecia control compared to the normal control (Figure 8 and Figure S1). The group treated with oral finasteride demonstrated 109%, 96.0%, 115%, and 9.93% higher expression levels of ALDH1, β-catenin, cyclin D1, and PKM2, respectively, as compared to the androgenic alopecia control, as well as 7.44%, 22.9%, and 4.8% greater expression of ALDH1, β-catenin, and cyclin D1, respectively, as compared to the 1% minoxidil solution-treated group (Figure 8 and Figure S1).

Treatment with AA in ethanol (1%) and AA–TF#15 (1%) induced higher protein expression of ALDH1, β-catenin, cyclin D1, and PKM2, compared to the groups treated with minoxidil (1%) and finasteride (1 mg/kg orally). The expression of ALDH1, β-catenin, cyclin D1, and PKM2 in the skin of AA–TF#15 (1%)-treated mice was highest amongst the treatment groups with 29.3%, 9.86%, 13.6%, and 25.4% higher expression levels than with finasteride (1 mg/kg orally) treatment, and 19.3%, 19.0%, 10.2%, and 3.19% higher than in the AA in ethanol (1%) treatment group, respectively (Figure 8 and Figure S1). Overall, these results indicate AA–TF#15 (1%) can effectively activate hair regeneration-promoting pathways, as compared to minoxidil (1%), AA in ethanol (1%), and finasteride (1 mg/kg orally).

## 4. Discussion

In this study, AA was selected as a naturally extracted phenolic compound from the *Stereocaulon japonicum* to treat androgenic alopecia, due to the different mechanisms of action it utilizes compared to medications already approved by the FDA. AA was screened for cellular proliferation in HaCaT and HDP cells and assessed for its ability to accelerate the telogen-to-anagen phase transitions. Altered expression levels of proteins related to hair regrowth were also investigated using an in vivo androgenic mouse model designed with an appropriate delivery system.

The efficacy of any drug depends on its availability in the desired area through the use of an appropriate delivery system. AA has low water solubility, and the aim was to deliver it to the desired hair follicle sites using a topical delivery system. Therefore, ethanol was selected based on its ability to maintain a stable AA–TF solution, as well as for its ability to enhance lipid fluidity near the polar region of intracellular lipids in the SC [38]. Due to the volatile nature of ethanol, it cannot maintain the solubility of the drug in the applied area, which can reduce the availability of the drug. For effective topical delivery, both initial phase permeation, as well as the ability to maintain the drug over time, are important for providing immediate therapeutic action [50,51]. Therefore, surfactants, such as propylene glycol, Transcutol P, isopropyl myristate, PEG 400, and oleic acid, which all allow for high solubility of AA, were proposed to maintain the drug in the solubilized form at the applied areas, as well as in the formulations even after the evaporation of ethanol.

The examination of individual solvents that can effectively deliver the drug to the applied area was performed by analyzing the permeability of AA across an artificial membrane, as well as in human skin. Based on both permeability results, ethanol enhanced the permeation of AA in the initial phase, which is likely to be due to the high solubility of AA in ethanol. Ethanol can also act through a lipid extracting or lipid chain mobility mechanism to create pore channels in both types of membrane [43]. Moreover, the ability to spread ethanol across the applied area can also improve the distribution of the drug and promote efficient permeation. Short-chain aliphatic alcohols, such as ethanol, are known to enhance the skin permeation of active substances in the applied area [52]. However, ethanol evaporates over time with the application of AA–TF, leading to precipitation of the drug and a reduction in permeability. Therefore, surfactant was added, which resulted in the partitioning of the drug in two phases.

During the first phase of partitioning, ethanol allows rapid permeation, while the second phase of drug partitioning is based on the solubility of the solvent with AA, as well as its own mechanistic processes, such as SC channels created through lipid extraction from the bilayer matrix, fluidization of the lipid bilayer, and changes in SC protein conformations [41,53,54,55]. In addition, the generation of a supersaturated drug on the applied area can lead to effective partitioning, which occurs after the evaporation of ethanol, and a concentration gradient of the drug is created with surfactants. That said, the permeability of AA using different solvents indicates AA–TFs have similar permeability in human and artificial membranes. However, the partitioning of the drug with some AA–TFs (i.e., AA–TF#2, AA–TF#3, and #14–19) was found to be different. These results may be due to the higher rate of interaction between ethanol, propylene glycol, and Transcutol P with the polyether sulfone layer, which allows the drug to reach layers of polyolefin-wide pores of the artificial membrane compared to oleic acid [56]. Another reason for the enhanced permeation on human skin by AA–TF#14–19 may be the enhanced interaction with heterogeneous, protein-rich corneocytes within intracellular lipids caused by the close packing of oleic acid, compared to ethanol, propylene glycol, Transcutol P, and PEG 400 [56]. The permeability data from both artificial and human skin (AA–TF#2–6, AA–TF#14–16, AA–TF#17–19) indicated an increase in AA permeability with increasing concentrations of oleic acid. AA–TF#15 was found to be superior in human skin compared to other formulations based on the initial phase of permeation and effective long-term partitioning, as well as elevated deposition on deep skin layers. Other mechanisms involving skin hydration may also contribute additively to enhanced permeation of AA–TF#15 across the human skin layer due to its larger volume of water compared to other AA–TFs [57].

The development of HDPs and other cells in the hair follicle, such as HaCaT, is an essential process for hair growth and follicle development, and control HDP and HaCaT cell proliferation is important when evaluating hair-growth treatments [58]. Indeed, treatment with the optimized sample AA–TF#15 (>10 µM AA equivalent) resulted in enhanced cellular proliferation of HaCaT and HDP cells, compared to treatment with AA–TF#1. The rate of scratch-wound recovery by AA in the AA–TF#15 treated cells was also higher, as compared to AA–TF#1. In addition, wound recovery after 20 h of incubation with AA–TF#15 treatment occurred at the fastest rate when compared to minoxidil in HaCaT cells and higher than that of minoxidil treatment in HDP cells. The enhanced cellular infiltration of AA observed with AA–TF#15 treatment, compared to AA–TF#1, may result from the presence of oleic acid, which can promote AA uptake in the cellular matrix by enhancing attraction and binding, as well as interactions with the lipid-bilayer in the cell membrane. This mechanism is thought to promote endocytosis and passive diffusion of oleic acid in the cytoplasm, which sufficiently activates various pathways responsible for cellular proliferation [59,60]. While the lower level of ethanol in AA–TF#15 compared to AA–TF#1 may also lead to less cellular toxicity and contribute to the cellular proliferation observed with AA–TF#15 treatment.

To determine whether the AA–TF#15 topical delivery system would induce hair regrowth with a once-daily topical application, topical, once-daily testosterone treatments were used simultaneously in the androgenic mouse model with AA in ethanol, AA–TF#15, and minoxidil, as well as oral finasteride. Testosterone has been reported to cause disorder in the anagen phase of the hair growth cycle in dermal papilla cells, as well as with factors related to cell proliferation [61,62]. However, the treatment of mice with AA in ethanol enhanced hair regrowth, compared to the normal and androgenic alopecia control. These results indicate AA may be a potent competitor to testosterone when binding to androgenic receptors in the hair follicle, preventing disruption of the normal hair cycle, such as miniaturization, shortening of the anagen phase, and lengthening of the kenogen phase [7]. Indeed, the enhanced topical delivery during the initial phase of AA–TF#15 treatment, as well as the maintained partitioning of AA over time, has demonstrated significant conversion of telogen to anagen phases and higher rates of hair regrowth, compared to treatment with AA in ethanol and minoxidil. However, further investigations are required to determine the optimum topical doses and drug concentration of AA–TF#15 for maximum hair regrowth efficacy in various alopecia animal models.

Unlike minoxidil, the inhibition of androgen receptors by AA may also enhance hair regrowth in androgenic alopecia with AA–TF#15 treatment. The conversion of the telogen phase to the anagen phase with AA–TF#15 treatment occurred sooner in those treated with finasteride. These findings may have resulted from more AA being available in the dermal of AA–TF#15-treated mice, as compared to oral finasteride treatment which utilizes first-pass metabolism and involves a high volume of distribution [63,64]. Finasteride can inhibit the conversion of testosterone to DHT, which only reduces the rate of hair loss. On the other hand, AA can competitively occupy and inhibit the binding site of testosterone and DHT to prevent further hair loss [7]. Therefore, AA–TF#15 was found to produce higher rates of telogen conversion to the anagen phase and to stimulate greater hair regrowth. These results were further confirmed when AA–TF#15-treated mice were found to have longer hair follicles, a higher weight of regrown hair, and larger numbers of hair follicles in H&E staining compared to minoxidil and finasteride treatment.

The transition of four different stages is mediated by various signaling pathways and is necessary for the effective development of hair. Testosterone leads to shorter anagen periods in hair follicles, which increases the duration of the telogen or catagen phase [10]. Many pathways can be activated for the transition of the catagen/telogen phase to the anagen phase. Wnt/β-catenin is important in the process of hair growth, as it enhances the expression of the β-catenin-mediated gene. It also targets genes, such as cyclin D1, which are essential for the proliferation, growth, and differentiation of dermal papilla cells and increase the size and number of hair follicles [10,58,65]. ALDH1 is also an important marker in stem cells that functions in follicle development and the hair growth cycle to facilitate self-protection, differentiation, and expansion [66,67,68,69].

A reduction in β-catenin expression and its target gene cyclin D1, as well as ALDH1, which is regulated by Wnt/β-catenin pathways, was observed after testosterone treatment. These results indicate reduced activation of the Wnt/β-catenin-mediated pathway, which may result in reduced hair regeneration after the catagen phase [70]. However, the levels of β-catenin, cyclin D1, and ALDH1 were found to be higher in AA–TF#15 (1%, *w*/*w*)-treated mice compared to mice treated with finasteride (1 mg/kg orally), minoxidil (1%, *w*/*w*), and AA in ethanol (1%, *w*/*w*).

PKM2 is a key enzyme in the glycolysis pathway that produces the necessary bioenergetics for the repeated cycles of self-renewal and differentiation required by stem cells. PKM2 was elevated during the transition to the anagen phase in hair follicles, and levels remained elevated in this phase, while lower levels were found in the androgenic mouse model [71]. A reduced level of PKM2 was observed in testosterone-treated mice, while elevated expression of this protein occurred in the androgenic mice, regrowing hair after treatment with minoxidil (1%, *w*/*w*), AA in ethanol (1%), and AA–TF#15 (1%).

Therefore, AA is highly efficient at hair regrowth because it acts as a specific anti-androgen and activates various pathways responsible for the self-renewal, differentiation, and development of hair follicles. In addition, the delivery of AA was enhanced with the AA–TF#15 vehicle system, which is composed of volatile and nonvolatile solvents, as well as oleic acid, which is an effective permeation enhancer. These components amplify the effects of AA in the treatment of androgenic alopecia. AA–TF#15 may therefore be one of the best alternative therapeutics for the treatment of androgenic alopecia.

## 5. Conclusions

In this study, we developed a solvent mixture system composed of volatile and non-volatile components for the effective delivery of AA to the deep layers of skin in the dermis. The optimum formulation (AA–TF#15) was identified for its ability to increase immediate permeation significantly and enhance the partitioning of the drug over time. AA–TF#15 displayed a 4.72-fold increase in permeability and a 6.70-fold increase in dermal deposition compared to AA in ethanol at 24 h. AA–TF#15 enhanced cell proliferation and improved scratch-wound recovery rate, which is comparable to minoxidil at the same molar concentration. In vivo studies also indicate elicited improvement in the rate of telogen conversion to anagen, as well as hair regrowth. More hair follicles, longer follicle lengths, and higher levels of Wnt/β-catenin pathway activation, which maintains regular hair growth, were observed with AA–TF#15 treatment compared to finasteride and minoxidil treatment. These findings suggest AA–TF#15 has the potential to be a potent topical anti-androgen therapy for androgenic alopecia.

## Figures and Tables

**Table 1:** Compositions of AA-loaded topical formulations.

Formulations (%, *w*/*w*)	AA	Propylene Glycol	Transcutol P	Isopropyl Myristate	Polyethylene Glycol 400	Oleic Acid	Ethanol	Water
AA–TF#1	1						49.5	49.5
AA–TF#2	1	9.9					44.6	44.5
AA–TF#3	1		9.9				44.6	44.5
AA–TF#4	1			9.9			44.6	44.5
AA–TF#5	1				9.9		44.6	44.5
AA–TF#6	1					9.9	44.6	44.5
AA–TF#7	1				8.3	8.3	41.2	41.2
AA–TF#8	1				15.2	7.6	38.1	38.1
AA–TF#9	1				26.4	6.6	33.0	33.0
AA–TF#10	1				29.7	0.5	49.5	19.3
AA–TF#11	1				29.7	1	49.5	18.8
AA–TF#12	1				29.7	1.5	49.5	18.3
AA–TF#13	1				29.7	2	49.5	17.8
AA–TF#14	1					5	39.5	54.5
AA–TF#15	1					9.9	39.5	49.6
AA–TF#16	1					19.8	39.5	39.7
AA–TF#17	1					5	49.5	44.5
AA–TF#18	1					9.9	49.5	39.6
AA–TF#19	1					19.8	49.5	29.7

## Data Availability

Not applicable.

## Supplementary Materials

The following supporting information can be downloaded at: https://www.mdpi.com/article/10.3390/pharmaceutics15020340/s1, Figure S1: Expression of ALDH-1, β-catenin, cyclin D1, and PKM2 proteins in the dorsal skin of mice measured by western blotting.
