# Supplementary material for: Topical Delivery of Atraric Acid Derived from Stereocaulon japonicum with Enhanced Skin Permeation and Hair Regrowth Activity for Androgenic Alopecia
Source: Pharmaceutics. 2023 Jan 19;15(2):340. doi: 10.3390/pharmaceutics15020340 (PMC9960134; doi:10.3390/pharmaceutics15020340)
Supplement: Supplementary file 1 [file pharmaceutics-15-00340-s001.zip › pharmaceutics-2151840-supplementary.pdf]

(a)

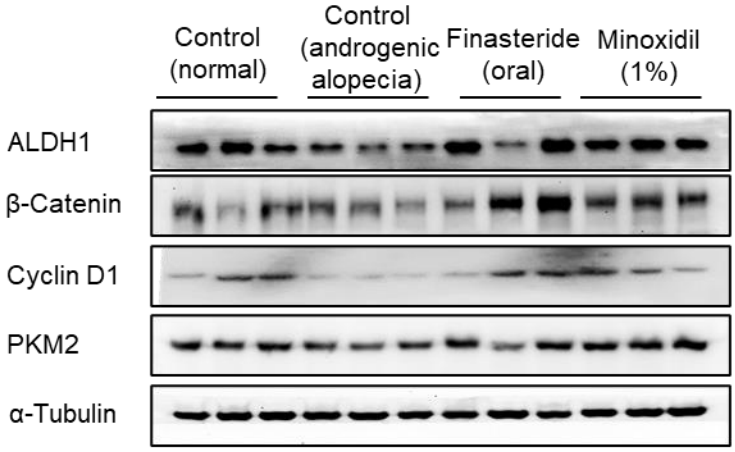

(b)

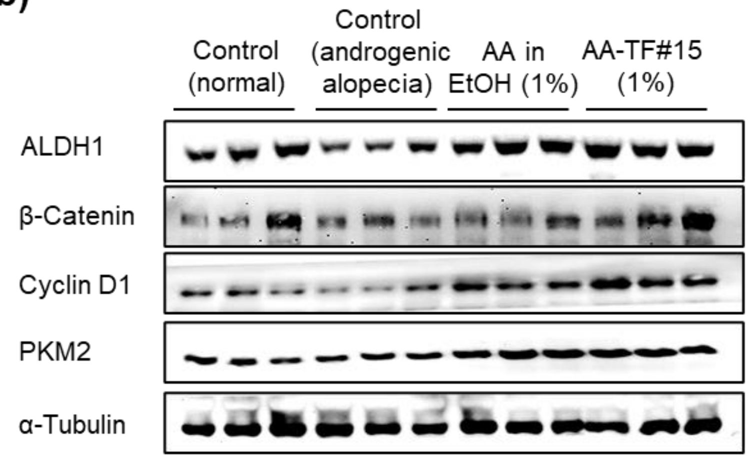

Figure S1. Expression of ALDH-1, β-catenin, cyclin D1, and PKM2 proteins in the dorsal skin of mice measured by western blotting. (**a, b**) Western blot analyses of ALDH-1, β-catenin, cyclin D1, and PKM2 proteins. Actin and *α*-tubulin are loading controls.
